# Evaluation of Performance and Longevity of Ti-Cu Dry Electrodes: Degradation Analysis Using Anodic Stripping Voltammetry

**DOI:** 10.3390/s24237477

**Published:** 2024-11-23

**Authors:** Daniel Carvalho, Ana Margarida Rodrigues, João Santos, Dulce Geraldo, Armando Ferreira, Marcio Assolin Correa, Eduardo Alves, Nuno Pessoa Barradas, Claudia Lopes, Filipe Vaz

**Affiliations:** 1 Physics Centre of Minho and Porto Universities (CF-UM-UP), University of Minho, 4710-057 Braga, Portugal; dani.fdcarvalho26@gmail.com (D.C.); armando.f@fisica.uminho.pt (A.F.); marciocorrea@fisica.ufrn.br (M.A.C.); fvaz@fisica.uminho.pt (F.V.); 2 Chemistry Centre, University of Minho, 4710-057 Braga, Portugal; anamaggy1989@gmail.com (A.M.R.); jpedro.fonsecasantos@gmail.com (J.S.); 3 LaPMET—Laboratory of Physics for Materials and Emergent Technologies, University of Minho, 4710-057 Braga, Portugal; 4 Graduate Program in Materials Science and Engineering, Federal University of Rio Grande do Norte (UFRN), Natal 59078-970, RN, Brazil; 5 Department of Physics, Federal University of Rio Grande do Norte (UFRN), Natal 59075-000, RN, Brazil; 6 Instituto de Plasmas e Fusão Nuclear, Instituto Superior Técnico, Universidade de Lisboa, Av. Rovisco Pais 1, 1049-001 Lisboa, Portugal; ealves@ctn.tecnico.ulisboa.pt (E.A.); nunoni@ctn.tecnico.ulisboa.pt (N.P.B.); 7 Centro de Ciências e Tecnologias Nucleares, Instituto Superior Técnico, Universidade de Lisboa, Estrada Nacional 10 Bobadela LRS, 2695-066 Lisboa, Portugal

**Keywords:** Ti-Cu dry electrodes, ASV, electrochemical performance

## Abstract

This study aimed to investigate the degradation of dry biopotential electrodes using the anodic stripping voltammetry (ASV) technique. The electrodes were based on Ti-Cu thin films deposited on different polymeric substrates (polyurethane, polylactic acid, and cellulose) by Direct Current (DC) magnetron sputtering. TiCu_0.34_ thin films (chemical composition of 25.4 at.% Cu and 74.6 at.% Ti) were prepared by sputtering a composite Ti target. For comparison purposes, a Cu-pure thin film was prepared under the same conditions and used as a reference. Both films exhibited dense microstructures with differences in surface topography and crystalline structure. The degradation process involved immersing TiCu_0.34_ and Cu-pure thin films in artificial sweat (prepared following the ISO standard 3160-2) for different durations (1 h, 4 h, 24 h, 168 h, and 240 h). ASV was the technique selected to quantify the amount of Cu(II) released by the electrodes immersed in the sweat solution. The optimal analysis conditions were set for 120 s and −1.0 V for time deposition and potential deposition, respectively, with a quantification limit of 0.050 ppm and a detection limit of 0.016 ppm. The results showed that TiCu_0.34_ electrodes on polyurethane substrates were significantly more reliable over time compared to Cu-pure electrodes. After 240 h of immersion, the TiCu_0.34_ electrodes released a maximum of 0.06 ppm Cu, while Cu-pure electrodes released 16 ppm. The results showed the significant impact of the substrate on the electrode’s longevity, with cellulose bases performing poorly. TiCu_0.34_ thin films on cellulose released 1.15 µg/cm^2^ of copper after 240 h, compared to 1.12 mg/cm^2^ from Cu-pure films deposited on the same substrate. Optical microscopy revealed that electrodes based on polylactic acid substrates were more prone to corrosion over time, whereas TiCu thin-film metallic glass-like structures on PU substrates showed extended lifespan. This study underscored the importance of assessing the degradation of dry biopotential electrodes for e-health applications, contributing to developing more durable and reliable sensing devices. While the study simulated real-world conditions using artificial sweat, it did not involve in vivo measurements.

## 1. Introduction

The demand for long-term care increases with the ageing population. Consequently, developing e-health physiological monitoring solutions becomes crucial to providing person-centred healthcare solutions and ensuring healthy lives and well-being for all ages. This necessity drives the development of durable, comfortable, and cost-effective physiological sensors, particularly in wearable technologies involving remote monitoring [1,2,3,4,5,6].

This study investigates the integrity of Ti-Cu dry biopotential electrodes intended for use in wearables for remote monitoring applications [1,7]. The electrodes were designed to be placed over the skin for long-term monitoring periods, which inevitably implies their contact with sweat, a very corrosive environment. Therefore, the impact of sweat on the electrode’s degradation and reliability over time was examined, drawing insights from earlier research [8]. The biopotential electrodes were prepared through magnetron sputtering by depositing nanometric Ti-Cu thin films onto different polymeric substrates: polylactic acid (PLA), polyurethane (PU), and cellulose. Biodegradable PLA was selected for its biocompatibility and biodegradability, making it ideal for skin-contact applications and minimising environmental impact. Cellulose was chosen for similar reasons, with its similarity to lyocell fabric enhancing the electrode’s integration into wearable systems. PU was selected for its flexibility, allowing easy conformity to the skin, and for its durability in harsh, high-sweat conditions. This selection of polymers with distinct characteristics and flexibility enables a comprehensive exploration of the electrode’s performance in wearable applications. The specific properties of each substrate lead to a complex relationship between the microstructure, morphology, and topography developed by the film [9], which are all critical characteristics of the electrode’s performance under real-world conditions. The degradation inflicted on the Ti-Cu biopotential dry electrodes can be easily assessed by electrochemical techniques. These techniques are distinguished by their ability to measure and control electrical quantities, offering a multifaceted characterisation of analytes, surpassing mere quantification. The versatility of electroanalytical techniques, particularly in providing selective, accurate, and highly sensitive results, positions them as indispensable tools across diverse domains, including environmental monitoring, industrial control, and biomedical analysis [10,11]. The essence of electrochemical techniques lies in measuring and controlling electrical quantities—current, potential, or charge—using an electrochemical cell typically consisting of three electrodes. Compared to other spectroscopic techniques, such as Atomic Absorption Spectroscopy (AAS), Inductively Coupled Plasma Mass Spectrometry (ICP-MS), and Inductively Coupled Plasma-Optical Emission Spectrometry (ICP-OES), electroanalytical techniques offer notable advantages in terms of selectivity and sensitivity, especially for the analysis of metal ions where pre-concentration steps can be performed (e.g., Cu, Sn, Zn, Cd, Au, or Cr) [10,12]. Furthermore, voltammetry techniques are both economical and suitable for integration into portable devices. This allows for real-time and continuous monitoring without the need for elaborate pretreatment of samples, making them an efficient choice for field applications and on-site analysis [13,14,15,16,17].

Voltammetric techniques measure current as a function of the potential applied to the working electrode, with cyclic voltammetry and pulse voltammetry techniques being the most widely used methods. Cyclic voltammetry involves dynamically altering the potential of the working electrode between two fixed values. This cyclic alteration allows for a comprehensive analysis of electrochemical systems, facilitating the study of redox compound behaviour and enabling the determination of reaction rate constants through detailed current–potential relationship data. Pulse voltammetry techniques, including Differential Pulse Voltammetry (DPV) and Square Wave Voltammetry (SWV), employ incremental potential steps, tailored to the specific potential profile of the working electrode. These techniques offer enhanced sensitivity and superior resolution compared to cyclic voltammetry, making them especially advantageous for analysing complex samples with low analyte concentrations [18].

Stripping voltammetry is distinguished for its ability to reach lower quantification limits. This is accomplished through a pre-concentration phase, where the analyte is first deposited on the electrode, followed by its dissolution. This process amplifies the measured current due to the increased analyte concentration at the electrode surface. This characteristic makes stripping voltammetry exceptionally suitable for detecting trace levels of metals in environmental and biomedical samples. Anodic stripping voltammetry (ASV), a subset of this technique, is known for its high sensitivity and minimal susceptibility to sample interference, yielding precise and reliable results even in complex matrices. It is particularly useful for determining heavy metal ions in biological solutions at trace levels [19,20,21,22]. For even lower detection limits, the stripping phase in ASV can be effectively combined with pulse techniques like DPV or SWV, harnessing their sensitivity to enhance the overall analytical performance [22,23,24].

Building upon a previous study that evaluated the lifespan of TiAg dry electrodes [8], with a particular focus on the role of ASV in understanding degradation dynamics, this research extends those findings to the Ti-Cu electrodes designed for biopotential monitoring. ASV is highlighted due to its unique ability to elucidate the complex degradation processes of Ti-Cu electrodes. Through systematic evaluation of Cu(II) released into sweat solutions over various periods, valuable insights into the durability and structural integrity of the dry electrodes are provided. Such insights are crucial, given copper’s susceptibility to corrosion in aggressive environments such as human sweat, which could potentially compromise the electrodes’ long-term functionality.

## 2. Materials and Methods

### 2.1. Preparation of the Ti-Cu Electrodes

The polymeric substrates for the electrodes were produced via 3D fused deposition modelling (FDM), using a ZMorph 3D Printer (Model VX printer Wrocław, Wrocław, Poland). Three different filaments with 1.75 mm diameter were selected: (i) biodegradable polylactic acid (PLA) (Eastman Amphora™ AM3300, Kingsport, TN, USA); (ii) flexible thermoplastic polyurethane (PU) (SMARTFILL, FLEX Lot Code 155264002953, Alcalá la Real, Jaén, Spain), and (iii) sustainable cellulose nanofibrils (FILAMENTIVE, Batch 840620, New Lane, Bradford, UK). The disc-shaped substrates (radius of 7.5 mm and height of 1 mm) were designed with a flat surface to be in contact with the skin and a snap button on top for the electrode’s mechanical and electrical connections into wearables. Further details can be found in [1]. The effective coated area of each electrode was determined as 433.10 mm^2^.

Immediately before the depositions, the polymeric substrates were cleaned with ethanol and activated through plasma treatments [7,25]. It is widely recognised that polymers exhibit low surface energy with reduced wettability, leading to challenging adhesion problems with metallic thin films [26]. However, plasma treatments in reactive atmospheres offer the possibility to significantly change the chemical and physical characteristics of the polymer’s surface, thereby promoting stronger adhesion at the polymer/thin film interface. During the plasma process, the polymeric substrate is activated by different mechanisms: (i) cleaning; (ii) etching; and/or (iii) activation promoted by the creation of new reactive sites. Plasma effectively removes organic contaminants and impurities from the surface (cleaning), modifies the surface texture/roughness through bombardment with energetic ions and reactive species (etching), and promotes the scission of the polymeric chains and the introduction of functional groups at reactive sites (activation), OH, -OOH, or dangling oxygen bonds for oxygen reactive atmospheres, as well as -NH_3_ or (R_2_C=NR) groups for nitrogen-rich atmospheres [1]. The optimal experimental parameters for plasma treatment, to activate PLA, PU, and cellulose substrates, were investigated in a previous work [1] and employed in the current study as outlined in Table 1.

The plasma treatments were conducted in a Diener plasma cleaner system (Plasma System Zepto, Diener electronic GmbH & Company KG, Ebhausen, Germany) equipped with a 13.56 MHz generator connected to a rotary pump operating at a low base pressure of 20 Pa.

After activation, the substrates were deposited by Direct Current (DC) magnetron sputtering. The samples were positioned on a three-dimensional grounded sample holder at the centre of the vacuum chamber. The depositions were carried out using a metallic target with 99.99 at.% of purity and dimensions of 200 × 100 × 6 mm^3^. Two different targets were used: pure Cu for the thin films used as a reference in this study, and a Ti target modified with 25 cylindrical pellets of Cu (area of 16 mm^2^ and thickness of 0.5 mm) glued onto its erosion zone for the preparation of the Ti-Cu thin films. The depositions were conducted by applying a current density of 75 A/m^2^ to the target, at room temperature and in rotation mode (5 rpm), to guarantee the integrity of the polymer’s characteristics and the homogeneity of the as-deposited electrodes. The deposition time was carefully adjusted to achieve a consistent thickness of approximately 200 nm. The base pressure of the chamber was kept below 7.0 × 10^−4^ Pa, while the working pressure was set to 3.0 × 10^−1^ Pa, using an Ar flow rate of 25 sccm.

### 2.2. Characterisation of the Ti-Cu Electrodes

The as-deposited Cu and Ti-Cu thin films were characterised in terms of their chemical composition, crystallinity, and morphology. To perform the (micro)structural analysis, silicon substrates (100 P-type, (100) orientation) were used.

The chemical composition of both Cu and Ti-Cu thin films was determined by Rutherford Backscattering Spectrometry (RBS). The measurements were conducted using a 2 MeV beam of ions ^4^He^+^ at normal incidence, with three detectors for the backscattered ions: one silicon surface barrier detector located at a 140° scattering angle and two-pin diode detectors symmetrically positioned relative to each other at 165°. The recorded data were analysed using the NDF software 10.1 [27,28].

The surface morphology and the growth microstructure of the films were examined through scanning electron microscopy (SEM) using a high-resolution microscope (NanoSEM-FEI Nova 200: FEI Company, part of Thermo Fisher Scientific, Hillsboro, OR, USA) equipped with a field emission gun. The cross-section micrograph analysis was used to determine the films’ thickness.

The assessment of the crystallinity and phase distribution of Ti-Cu thin films was carried out through X-ray diffraction (XRD), using a Bruker D8 diffractometer (Bruker Corp., Billerica, MA, USA). The analysis was conducted in grazing incidence geometry with Cu-Kα radiation (λ = 1.5406 Å), maintaining the step size of 0.04° within the 2θ range from 25° to 90°.

Additionally, the optical surface characteristics of the as-deposited and corroded electrodes were observed in detail using a Dino-Lite digital microscope (AM7013MZT4: AnMo Electronics Corporation, New Taipei City, Taiwan) with 5-megapixel resolution and 450× magnification. For each sample, four different photographs were taken (immediately before and after immersion in artificial sweat for different periods). Each captured image was analysed by MATLAB (version R2018a, The MathWorks, Inc., Natick, MA, USA), producing binary images through a thresholding approach to evaluate the optical defects promoted on the surface of the electrode by corrosion processes [29].

### 2.3. Degradation of the Ti-Cu Electrodes

Considering their intended real-world application involving prolonged contact with sweaty skin, Ti-Cu dry electrodes were subjected to immersion in artificial sweat to assess their degradation over time. Sweat, known for its corrosive nature, has the potential to corrode the metallic thin films, thereby impacting the electrodes’ performance [30]. Each type of electrode, consisting of the same thin film and polymer, was immersed into 15 mL of a physiological sweat solution prepared according to ISO standard 3160-2 [31].

All electrodes were placed in an incubator (LabCompanion IST-4075R) regulated at body temperature (37.0 ± 0.1 °C), with a rotation speed set at 30 rpm, for durations of 1 h, 4 h, 24 h, 168 h, and 240 h. The impact of sweat degradation on the electrodes was evaluated by measuring the amount of Cu(II) released into the sweat solution using ASV. Two electrodes from each condition were analysed for comparative purposes.

### 2.4. ASV Analysis

**Figure 1:**
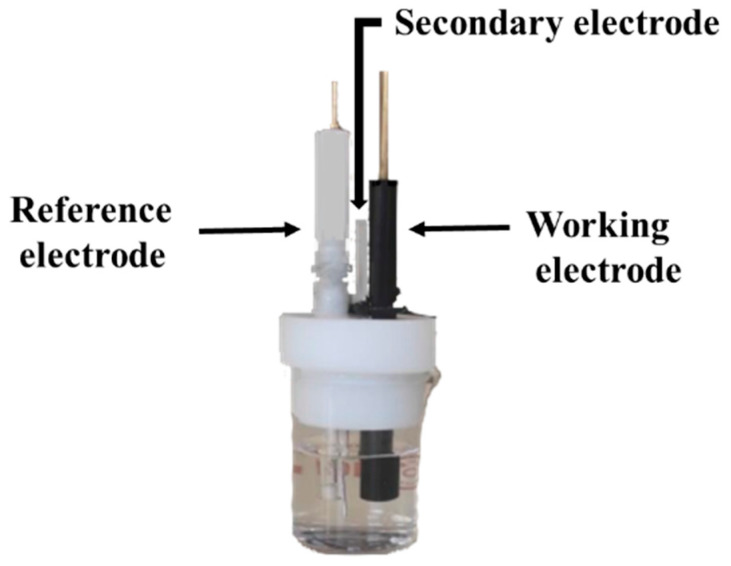
Electrochemical cell used for the ASV experiments.

Anodic stripping voltammetry (ASV) was performed using an Autolab potentiostat (PGSTAT30, Ecochemie: Eco Chemie B.V., Utrecht, The Netherlands) controlled by GPES 4.9 software. The experiments were performed in a three-electrode cell, comprising (i) an Ag/AgCl reference electrode (1 M KCl; CH Instruments Inc.: IJ Cambria Scientific Ltd., Llanelli, UK); (ii) a secondary platinum wire electrode (CHI115 CH Instruments: IJ Cambria Scientific Ltd., UK); and (iii) a glassy carbon working electrode (GCE; 3 mm diameter; BAS M-2012: West Lafayette, IN 47906, USA), as illustrated in Figure 1. Before the voltammetric experiments, the GCE was polished with alumina powder (0.05 μm; Bulher: Lake Bluff, IL, USA) on a polishing cloth. All electrodes were carefully rinsed with ultrapure water and dried with absorbent paper before each voltammogram recording. Immediately after the experiments, the reference electrode was washed and immersed in a 1 M KCl solution.

Voltammograms were acquired using the Square Wave Voltammetry (SWV) technique, following established parameters from the literature [32]: frequency of 80 Hz, pulse amplitude of 100 mV, and potential step of 2 mV. All experiments were conducted at room temperature (25 ± 2 °C) with stirring set at 300 rpm for the deposition step and an equilibrium time of 5 s.

## 3. Results

### 3.1. Thin Film Characterisation

The use of Ti-Cu thin films as biopotential electrodes has been investigated in previous work [1]. The chemical composition revealed that the thin films prepared with a Ti-composed target, containing 25 pellets glued on the erosion zone, resulted in a Cu/Ti chemical proportion of 1:3. The films were composed of 25.6 at.% Cu and 74.4 at.% Ti, yielding a Cu/Ti atomic ratio of 0.34 (TiCu_0.34_) in contrast to the pure-Cu films used as reference and prepared under the same conditions. The RBS results were also fundamental for determining the films’ densities, which were found to be approximately similar, despite the Cu film having a slightly higher density (9.27 × 10^22^ at/cm^3^), compared to 8.02 × 10^22^ at/cm^3^ for the TiCu_0.34_ thin films.

**Figure 2:**
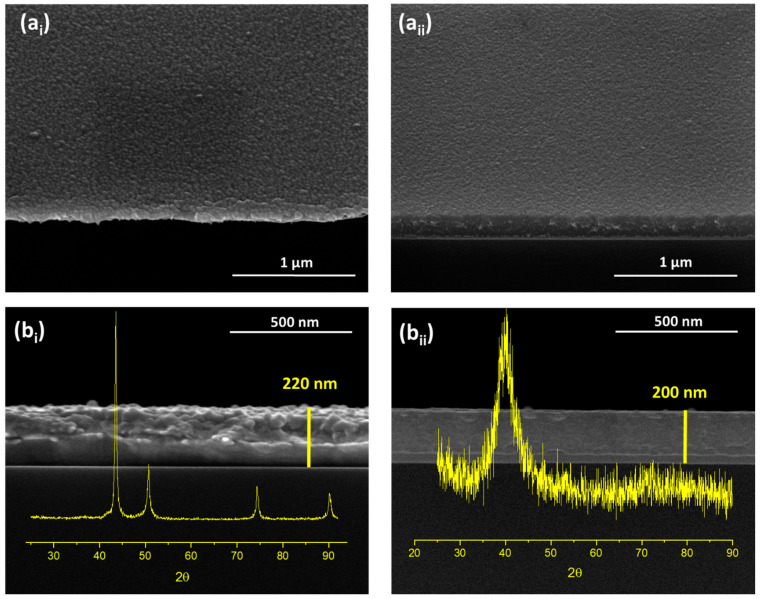
SEM images representative of (**a**) the surface morphology and (**b**) cross-section view of the film’s growth with the respective X-ray diffractograms for (**i**) Cu-pure film and (**ii**) TiCu_0.34_ thin film.

The microstructure of the TiCu_0.34_ thin films was analysed with the Cu-pure thin film as a reference. Figure 2 shows the topography and cross-section micrographs of both films, as well as their crystalline structures.

Compared to the Cu-pure thin film (Figure 2a_i_), the TiCu_0.34_ thin films (Figure 2a_ii_) exhibited a smoother surface with a fine-grained microstructure, which is a common feature observed for Ti-based binary systems [1,7,8]. Regarding the microstructure, there is no evidence of columnar growth; instead, both films prepared with similar thicknesses (≈200 nm) revealed very dense and featureless microstructures, consistent with the high atomic density determined by RBS.

The Cu-pure reference film displays the typical microstructure of ductile materials, characterised by a high-density compact growth and a well-defined atomic face-centred cubic (fcc) structural arrangement. As shown in Figure 2b_i_, the XRD pattern of the Cu-pure film evidenced a well-defined fcc polycrystalline structure, indicating the absence of significant voids or defects in the films’ structure [33,34]. Four distinct Bragg peaks are noticeable at the positions 43.56°, 50.72°, 74.36°, and 90.24°, corresponding to the (111), (002), (022), and (113) orientations, respectively, according to the ICSD card #426938. Moreover, the cross-sectional image of the as-deposited Cu thin film brings some evidence of poor adhesion to the substrate. Several studies have reported the significant influence of residual stresses on the extent of plastic deformation during debonding processes of ductile thin-film structures on brittle substrates [35,36,37]. This phenomenon was particularly studied in Cu thin films used in electronic devices. The sputtering discharge parameters, including target potential, substrate temperature, and the use of substrate biasing, primarily influence residual stresses. The energy of the impacting particles is also crucial for creating implantation sites on the substrate’s surface, thereby improving interfacial adhesion. However, in this work, due to the specific characteristics of the substrates, the depositions were carried out at room temperature without BIAS, reducing the ballistic energy of the sputtered Cu atoms impacting the substrate and compromising adhesion. Additionally, the high deposition rate of the reference Cu-pure film (0.9 nm/s), which was 3.6 times higher than the deposition rate of the TiCu_0.34_ thin film, could affect the surface diffusion of the Cu ad-atoms, contributing to reduced adhesion [38,39].

In contrast, the TiCu_0.34_ thin film, despite its dense microstructures (Figure 2b_ii_), revealed visible shear striations and vein patterns, typical features of thin-film metallic glasses (TFMGs). This evidence was confirmed by the amorphous diffraction pattern exhibited by the TiCu_0.34_ thin film, with a broad diffraction hump at 2θ ranging from 35° to 47°, indicating the film’s amorphous structure. This suggests the presence of one or more metastable Ti-Cu intermetallic phases [1,7,40], precipitating in minor traces, below the XRD limit detection. Unlike the crystalline Cu-pure film of reference, the disordered arrangement shown by the TiCu_0.34_ thin film and the absence of crystalline grains led to the smoother surface morphology observed in Figure 2a_ii_, which is also a typical feature of TFMGs [1,7,41].

In fact, the atomic density determined for the TiCu thin films by RBS measurements shows a high atomic packing density of the film, increasing from 5.62 × 10^22^ at/cm^3^ (theoretical calculation for a pure-Ti hexagonal close-packed structure) [42,43] to 8.02 × 10^22^ at/cm^3^, close to that of the Cu thin film. High atomic packing density is also a characteristic of amorphous metallic structures forming glassy structures [44,45,46]. As the Cu dopant element (atomic radii 128 pm) is smaller than the Ti matrix element (atomic radii 146 pm), it can easily occupy interstitial sites or substitute matrix atoms in the hcp Ti structure. According to the model developed by O.N Senkov and D.B. Miracle for bulk amorphous alloys [42], interstitial and substitutional atoms attract each other, producing short-range order atomic clusters. These clusters stabilise the amorphous state and create glassy structures, which seems to be evident in the behaviour of the TiCu_0.34_ thin films.

### 3.2. Voltammetry Analysis

#### 3.2.1. ASV Optimisation

**Figure 3:**
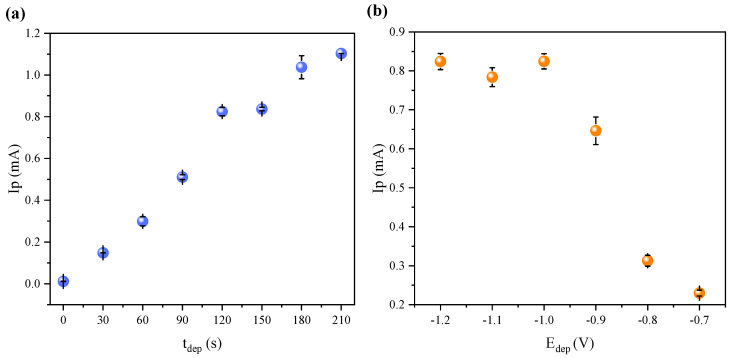
Variation in the peak current (Ip) of a 5.00 ppm solution of Cu(II) in artificial sweat as a function of (**a**) deposition time (t_dep_) at a deposition potential of −1.0 V and (**b**) deposition potential (E_dep_) with a deposition time of 120 s. Data were obtained using the ASV-SWV technique with the established parameters.

The experimental parameters for the anodic stripping voltammetry coupled with square wave modulation (ASV-SWV) were refined to accurately quantify trace amounts of Cu(II) in artificial sweat following electrodes’ immersion, aiming to develop a more sensitive method. Consequently, the optimal parameters of deposition time (t_dep_) and deposition potential (E_dep_) were investigated using a 5.00 ppm solution of Cu(II) prepared by diluting a Cu(II) stock solution (1000 ppm; Merck Company: Merck Certipur—Darmstadt, Germany) in artificial sweat. For the t_dep_, a range of 0 to 210 s with 30 s intervals was tested, while maintaining E_dep_ at −1.0 V [22,47]. Regarding the E_dep_, potentials ranging from −1.20 V to −0.70 V with increasing increments of 0.10 V were evaluated, with t_dep_ fixed at 120 s. Each test was conducted in triplicate to ensure precision and reliability. The obtained results are depicted in Figure 3.

As expected, the peak current (Ip) increased with the t_dep_, as shown in Figure 3a. Longer deposition times allow for a greater deposition of Cu(II) ions on the working electrode, consequently yielding higher peak currents during the subsequent stripping step. Despite the higher Ip values associated with longer deposition durations, the t_dep_ of 120 s was selected to determine the release of Cu(II) ions by the Ti-Cu electrodes. This choice was made considering that it enables fast analyses, and longer times do not properly benefit the Ip/t_dep_ ratio.

Conversely, the extension of the electrochemical reduction decreases as the deposition potential E_dep_ becomes less negative (Figure 3b). More negative E_dep_ values promote higher rates of Cu(II) ion reduction, resulting in greater deposition on the electrode surface and consequently higher peak currents during the stripping step. The Ip value reached its maximum at a potential of −1.0 V, which was selected as the optimal deposition potential for all subsequent tests performed on the electrodes.

#### 3.2.2. Determination of Cu(II) into Artificial Sweat

**Figure 4:**
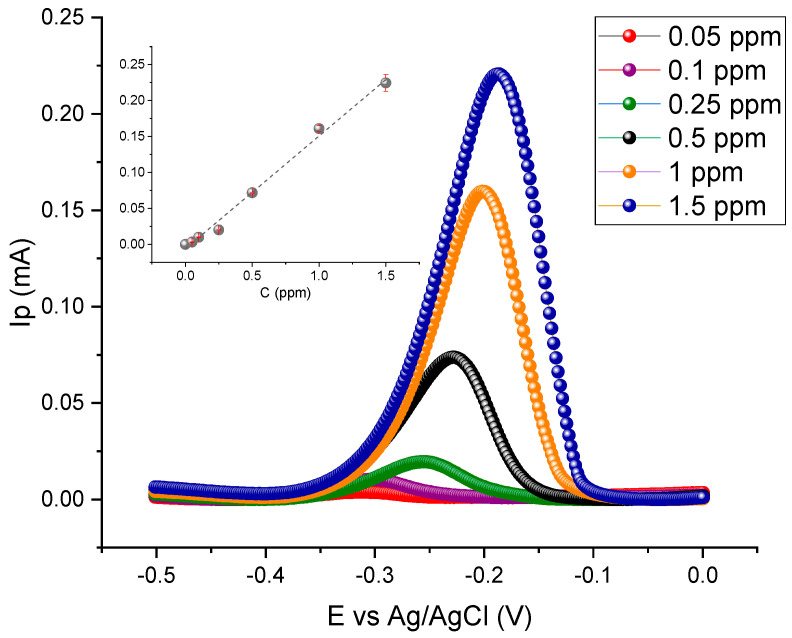
Anodic stripping voltammograms of copper in artificial sweat obtained at the GCE electrode for the standard Cu(II) solutions with different concentrations. To ascertain the limit of quantification (LOQ) and the limit of detection (LOD) of the method, 6 replicate analyses of the standard Cu(II) solution with the lowest concentration (0.05 ppm) were performed. The relative standard deviation was less than 10%, satisfying the acceptance criterion, thus establishing the LOQ at 0.05 ppm. The LOD was estimated to be one-third of the LOQ, yielding a value of 0.016 ppm. Although this LOD is relatively low, it is higher than the values reported in the literature (0.0002 ppm) [22]. This suggests that while the current method is effective, there is still room for improvement.

After setting the fundamental parameters for the optimisation of ASV-SWV technique, a calibration curve was obtained by varying the concentration of Cu(II) from 0.050 to 1.5 ppm. Standard solutions were prepared through the Cu(II) stock solution referred to in Section 3.2.1. Figure 4 shows the voltammograms obtained using ASV-SWV for the standard solutions, with the inset featuring the respective calibration curve, delineating the relationship between copper concentration and peak current response.

Figure 4 evidences a linear relationship between the stripping peak current and Cu(II) concentration in the range of 0.05 to 1.50 ppm. The calibration curve is described by Equation (1), with uncertainties calculated using Student’s t-factor for a 95% confidence level with four degrees of freedom.

(1) $$I_{p}\left(mA\right)=\left(0.16\pm 0.02\right)C\left(ppm\right)-(0.01\pm 0.01)$$

The correlation coefficient of 0.996 confirms the linearity of the method. Thus, the calibration curve described by Equation (1) was used to quantify the Cu(II) released by the Ti-Cu electrodes immersed in artificial sweat solutions during distinct periods.

### 3.3. Analytical Assessment of Ti-Cu Electrodes in Artificial Sweat

**Figure 5:**
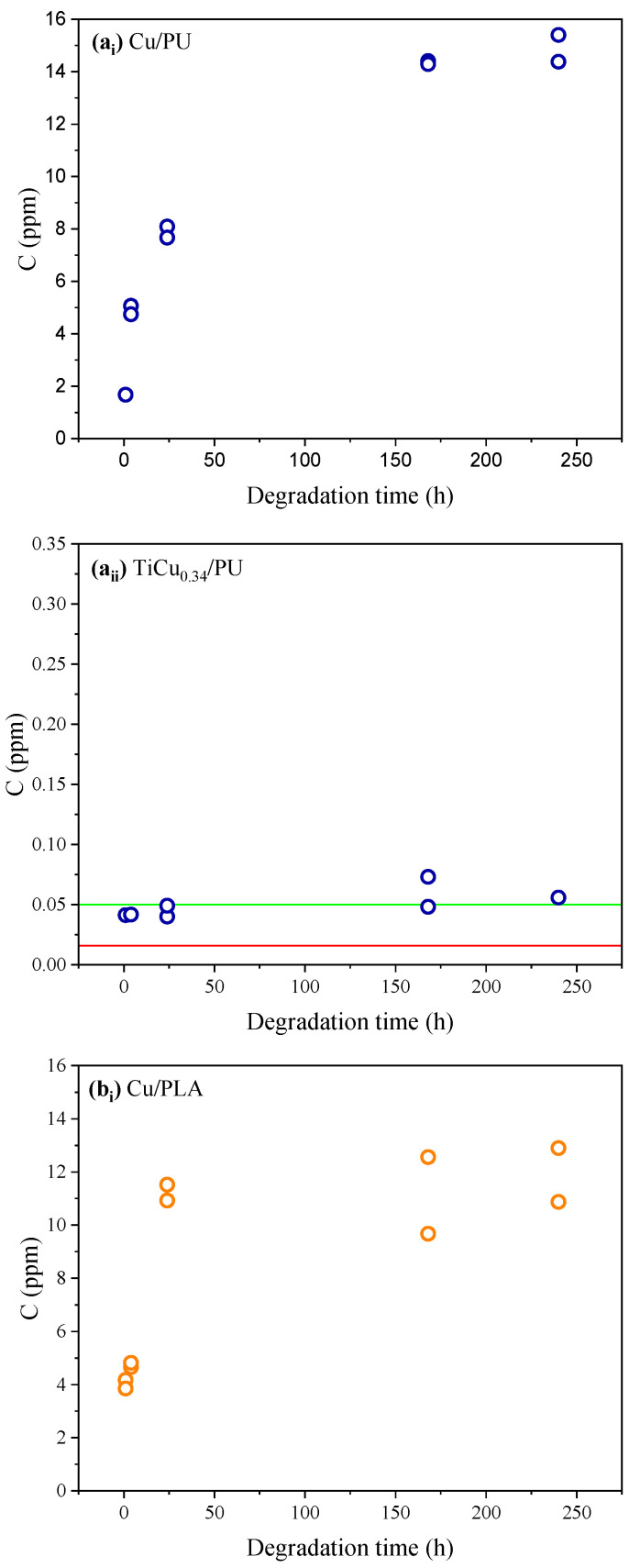

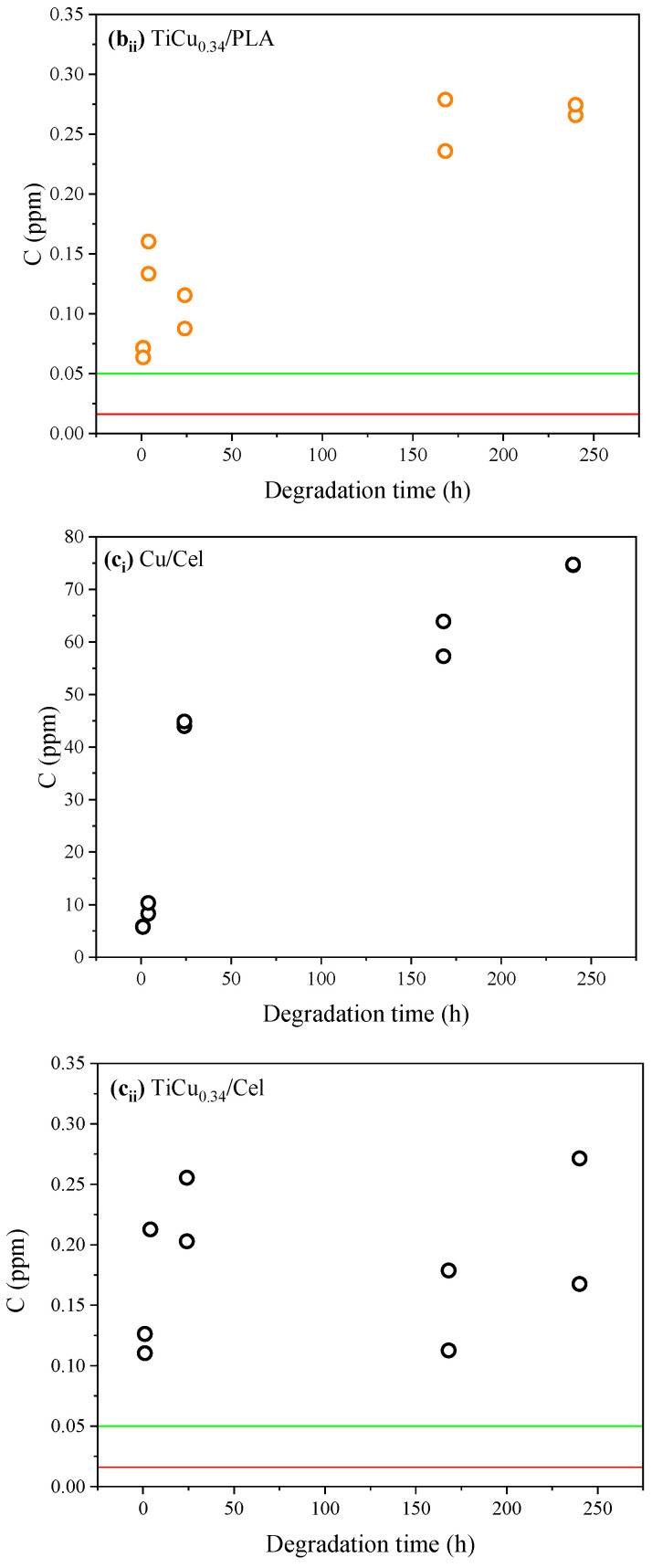
Copper concentration determined in artificial sweat released from the electrodes of (**i**) Cu-pure and (**ii**) TiCu_0.34_, immersed in artificial sweat using different substrates: (**a**) PU, (**b**) PLA, and (**c**) cellulose. The red line represents the LOD (limit of detection), and the green line represents the LOQ (limit of quantification).

After establishing the calibration curve under optimised conditions, the copper concentration was determined in 15 mL of the artificial sweat samples where the Ti-Cu electrodes underwent degradation. As this study aims to assess the impact of sweat on the degradation of the TiCu_0.34_ electrodes prepared with different polymeric substrates (PLA, PU, and cellulose), two replicas of each type of electrode were used. For comparison, electrodes based on Cu-pure films deposited on the same substrates and submitted to the same degradation conditions were also tested. The results are depicted in Figure 5. Despite the same trend, in some cases, it is possible to note some discrepancies between the replicas. Since the polymeric bases were 3D-printed, the possibility of significant structural variations in the substrates must be considered. These variations can affect the deposition of the thin film and, consequently, the final performance of the electrode.

The concentration of Cu(II) released by the TiCu_0.34_ electrodes into artificial sweat is approximately three times lower than that released from Cu-pure electrodes (reference), across all the substrate types, under the same degradation conditions. The superior performance of the TiCu_0.34_ electrodes is closely related to the thin films’ characteristics discussed in Section 3.1. In fact, the corrosion behaviour is strongly influenced by the morphology of the thin film [48].

The dense and featureless microstructure of the TiCu_0.34_ thin film, typical of TFMGs, results in smoother surfaces free from defects, maintaining the electrode’s integrity over extended periods in corrosive environments. Additionally, Ti is well known for its impressive corrosion resistance due to the formation of a stable passivation layer of titanium dioxide (TiO_2_) on its surface. Therefore, both the microstructure and the TiO_2_ passivation layer in the film containing almost 75 at.% of Ti could play a crucial role in resisting degradation over time [44,49]. In contrast, the poor performance of the Cu-pure electrodes, used as a reference, indicates their susceptibility to dissolution. The Cu thin film, when exposed to the saline and corrosive environment of sweat, readily oxidises, leading to the formation of the cupric ion Cu(II). As a consequence, the rate of the copper released increases, which is a prime factor for its dissolution [49,50,51]. Additionally, the active–passive corrosion behaviour, along with the ductile microstructures discussed in Section 3.1, further weakens the poor adhesion of the film to the substrate, facilitating corrosion.

The release of Cu(II) from the TiCu_0.34_/PU electrodes (Figure 5a_ii_) remains very low and relatively constant, regardless of the degradation time. For degradation periods of less than 24 h, the concentration of Cu(II) released remains under the LOQ, and after 240 h immersed in artificial sweat, it does not exceed 0.06 ppm. The result suggests that no significant degradation occurs for these electrodes after the first hour of contact with artificial sweat. In turn, for the reference Cu-pure electrodes, the concentration of Cu(II) in the artificial sweat solution significantly increases from approximately 2 to 8 ppm in the first 24 h to reach the maximum value of 15.4 ppm after 240 h. The results leave no doubts about the severity of the corrosion processes in the electrodes of pure Cu.

Except for the TiCu_0.34_/PU electrodes, the integrity of all other electrodes prepared in this work highly depends on the degradation time. For TiCu_0.34_ electrodes based on PLA (Figure 5b_ii_), the amount of Cu(II) released nearly doubles (from 0.07 to 0.16 ppm) after 24 h, always exceeding the LOQ. There is a clear increase in the amount of metal released into the sweat solution over time, showing a tendency to stabilise from 168 h onwards. In turn, the reference electrodes using PU substrates (Figure 5b_i_) exhibit a similar behaviour to that reported for TiCu_0.34_/PU electrodes. After 24 h, the Cu(II) concentration in the solution exceeds 8 ppm, exhibiting a constant Cu(II) dissolution for longer immersion times.

For TiCu_0.34_ electrodes based on cellulose (Figure 5c_ii_), the Cu(II) concentration released into the solution doubles (from 0.11 to 0.21 ppm) after the first 4 h of immersion in artificial sweat solution. After the first hour of immersion, the Cu(II) released by these electrodes was already over the LOQ. This behaviour significantly worsens for Cu-pure electrodes (Figure 5c_i_) where the results seem to indicate that for longer periods (>168 h), the entire thin film was dissolved and released to the sweat solution, due to the high concentrations detected (≈75 ppm).

**Figure 6:**
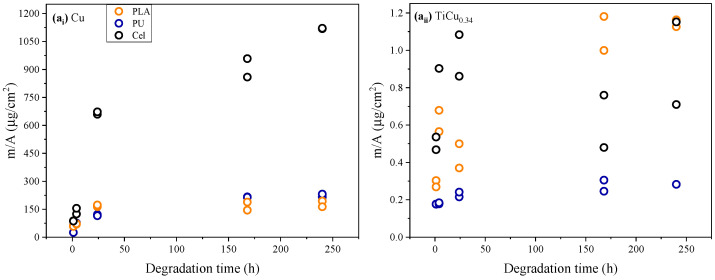

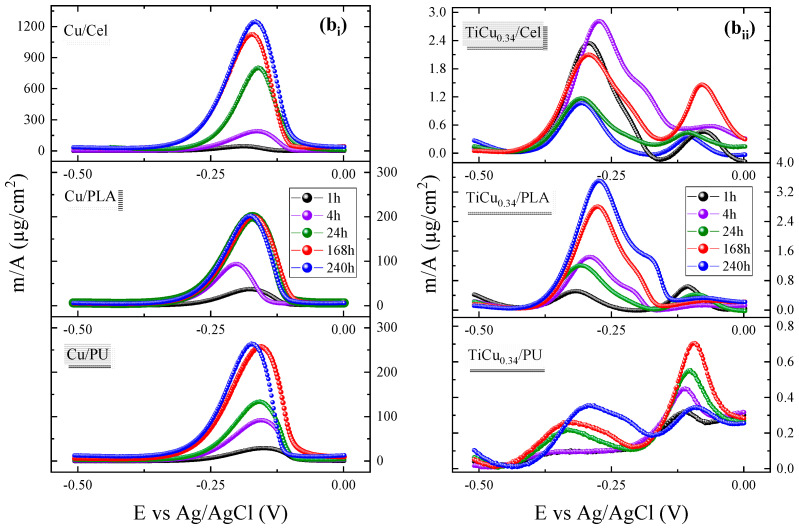
Mass of copper released per unit area in the artificial sweat solution from thin films of (**a_i_**) Cu-pure and (**a_ii_**) TiCu_0.34_ deposited on the different substrates, along with the experimental voltammograms used to determine the cooper release for (**b_i_**) Cu-pure electrodes and (**b_ii_**) TiCu_0.34_ pure electrodes.

The results clearly demonstrate the significant influence of the substrate on the electrode’s degradation. To facilitate a comprehensive analysis, the concentration of Cu(II) in the sweat solution was converted to mass released per unit area (µg/cm^2^), considering the active area of the electrodes. Figure 6 enables a comparison of the substrate’s impact on the degradation behaviour of Cu-pure and TiCu_0.34_ electrodes over time.

Cellulose substrates have the greatest negative impact on electrode performance. This effect is particularly evident for Cu-pure electrodes but is also consistent for TiCu_0.34_ electrodes. Except during the 168 h immersion period, when TiCu_0.34_/PLA electrodes show the poorest performance, the high porosity of cellulose significantly affects the characteristics and properties of the deposited films, reducing the electrode’s lifetime. Also, for the Cu-pure electrodes on cellulose, the copper mass determined in sweat reaches milligrams per cm^2^ levels of 1.12 mg/cm^2^ after 240 h of immersion.

The results also show that, even if the amount of Cu(II) in the TiCu_0.34_ electrodes were four times greater (100 at.% of Cu as in the Cu-pure film), the release would not exceed 5.00 µg/cm^2^ (after 240 h of immersion). This value is still lower than the smallest amount released by the Cu-pure electrodes just 1 h after degradation (25 µg/cm^2^), underscoring the quality of the TFMG-like microstructure developed in the TiCu_0.34_ film, which hinders sweat penetration and preserves the electrode integrity for extended periods.

Considering all the results, TiCu_0.34_ electrodes prepared with PU substrates emerge as the preferable option for real applications. The amount of copper released to sweat was the lowest, under or close to the LOQ, with no significant variation over time. After 240 h, only 0.28 µg/cm^2^ of copper was released into the sweat solution, indicating the high resistance of these electrodes to sweat-induced degradation and an improved lifespan. In comparison to Ti-Ag dry electrodes deposited on PTFE substrates, analysed by the authors in a previous study [8], the TiCu_0.34_ electrodes demonstrate greater stability. For similar chemical compositions (TiAg_0.23_ electrodes), a higher release rate of Ag was detected, suggesting that TiCu_0.34_ electrodes may be more suitable for prolonged applications.

However, understanding the poor performance of TiCu_0.34_/PLA electrodes over extended periods is crucial. Therefore, the electrodes were subjected to optical characterisation immediately before and after immersion. Due to the specific experimental parameters and mechanisms involved in the sputtering process, there is a deep connection between substrate characteristics and film growth, a factor that strongly conditions the final properties of the electrode and amplifies the substrate defects. Since the polymeric substrates were prepared using 3D FDM, they inherently exhibit significant surface defects, including heterogeneities in surface topography, voids, porosity, and roughness. These defects are replicated and amplified during film deposition, increasing the susceptibility to degradation.

### 3.4. Optical Characterisation

**Figure 7:**
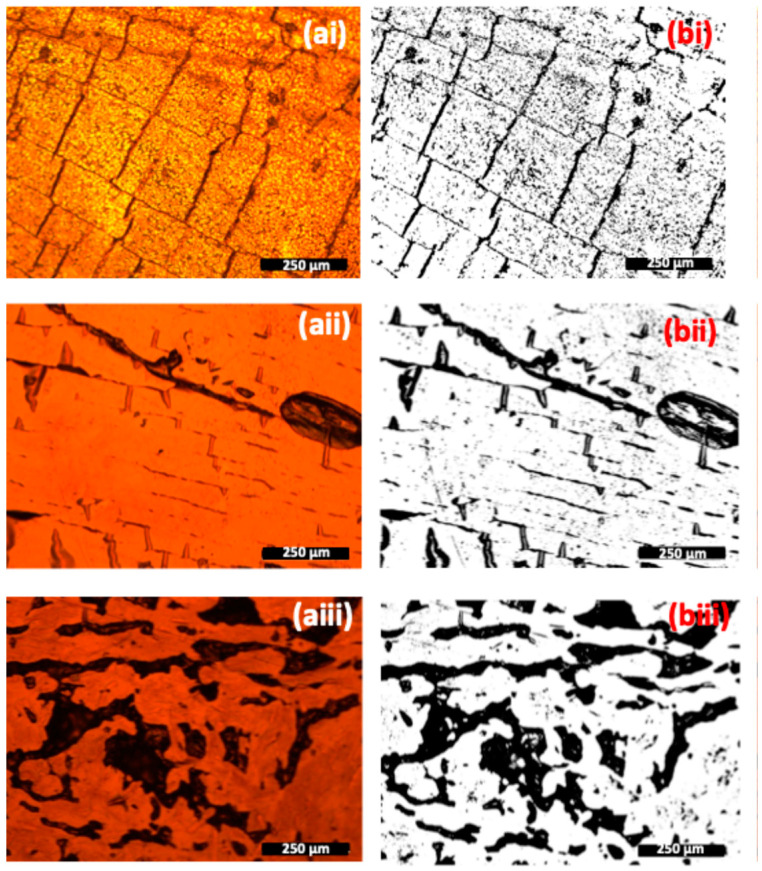
Optical characterisation of a (**i**) TiCu/PU, (**ii**) TiCu/PLA, (**iii**) TiCu/cellulose electrode’s surface before degradation: (**a**) are the images acquired by the Dino-Lite digital microscope and (**b**) the respective binary images, processed by MATLAB to calculate the optical defects (black areas in the image).

Optical characterisation helps understand the influence of defects on the degradation process and how the electrode/thin film surface evolves after various periods of immersion in physiological sweat. Surface defects on the electrode provide open paths for the electrolyte’s entry through the film, increasing corrosion kinetics. For the optical analysis, four images of each electrode’s surface were taken before and after sweat immersion. Figure 7 shows the surface of three randomly selected TiCu_0.34_ electrodes, each deposited on a different substrate. Images were captured with an optical microscope (Figure 7a), along with corresponding images created by a MATLAB algorithm, which quantifies the area of imperfections/heterogeneities marked by the dark field (Figure 7b).

**Figure 8:**
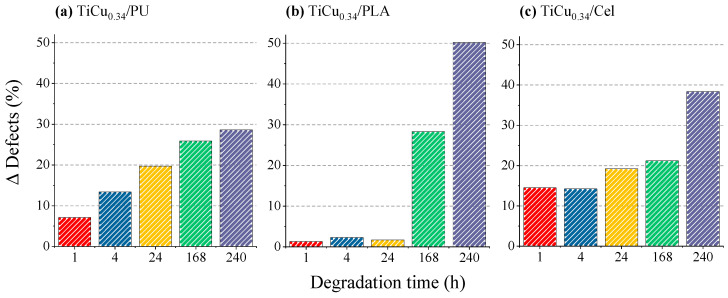
Variation in the number of defects on the surface of the TiCu electrodes prepared by the functionalisation of different polymeric bases (**a**) PU, (**b**) PLA, and (**c**) cellulose for 1 h, 4 h, 24 h, 168 h, and 240 h of degradation.

The optical pictures provide a measure of the defects’ progression on the film surface during degradation, emphasising the influence of the substrate on the film’s topography and on the overall behaviour of the electrode. The percentage of the damaged area is calculated by dividing the damaged area (black regions) by the total area of the image (Equation (2)). Since four images of each electrode’s surface were taken, the number of defects was given by the average number obtained for all images. The results, presented in Figure 8, show the defect’s progression as a function of the degradation time, where

(2) $$∆Defects\;(\%)=Defects\;\left(\%\right)\left(after\;degradation\right)-Defects\;\left(\%\right)(before\;degradation)$$

The homogeneities in the surface topography (Figure 8) of the electrode correlate closely with the concentration of Cu(II) released into the artificial sweat solution during different immersion periods (Figure 6). For the TiCu_0.34_/PU electrodes, a slow and gradual increase in heterogeneities is observed over extended immersion periods, indicative of a light degradation process (Figure 8a). This behaviour is similarly noted in the TiCu_0.34_ electrodes based on cellulose substrates during shorter degradation periods (Figure 8b). However, after 240 h, the percentage of defects significantly increases, reflecting the higher Cu(II) amount released into the sweat solution, as shown in Figure 6b. The most notable deviation is observed with the TiCu_0.34_/PLA electrodes. Here, the percentage of defects rises sharply during prolonged immersion periods (168 h and 240 h), highlighting the impact of the electrolyte on the electrode surface. As a consequence, the degradation of the film is accelerated leading to a higher release of Cu(II) content into the sweat solution.

The results for Cu electrodes are not presented, since after 1 h of immersion in physiological sweat, almost all the thin films had been released from the polymer into the solution, regardless of the type, due to dissolution phenomena promoted by corrosion processes. This evidence extensively confirms the poor resistance of the Cu-pure reference electrodes to degradation processes, shortening their service life. The active dissolution of Cu into sweat solutions has already been reported in several studies [49,50,51]. The anodic reaction process is accelerated by the sodium chloride (NaCl) in the sweat solution, resulting in the formation of reactive and soluble dichlorocuprate(I) ($Cu{Cl}_{2}^{-}$) ions, as well as cuprous oxide (Cu_2_O) and copper chloride hydroxide (Cu_2_(OH)_3_Cl), through which the Cu ions diffuse [50].

## 4. Conclusions

This study emphasises the critical importance of assessing the lifespan of dry biopotential electrodes, which have significantly contributed to the expansion of e-health applications in recent years. Based on the strong performance of Ti-Cu electrodes, this work evaluated their degradation and corrosion resistance using anodic stripping voltammetry. The study compared Ti-Cu thin film-based electrodes deposited on three polymeric substrates (PU, PLA, and cellulose) with Cu-pure electrodes, all prepared under identical conditions. The primary focus was on quantifying the degradation of thin-film electrodes by monitoring copper release into an artificial sweat solution over various immersion times.

The ASV analysis showed that TiCu_0.34_ electrodes released significantly less copper compared to Cu-pure electrodes. The type of substrate polymer had a substantial impact on the overall performance of the electrodes, with TiCu_0.34_/PU electrodes exhibiting exceptional performance. Notably, after 1 h of immersion, Cu-pure electrodes released approximately 2 ppm of Cu(II), which was 25 times higher than the 0.06 ppm released by TiCu_0.34_ electrodes, even after 240 h. The influence of the substrate on the electrode’s performance was also evidenced by the TiCu_0.34_ electrodes prepared with PLA and cellulose substrates, showing contrasting behaviours for different immersion times.

The findings indicate that the degradation of the electrodes is closely linked to the microstructural features of the thin films prepared by sputtering. The TFMG-like characteristics of the TiCu_0.34_ thin films, along with the excellent corrosion resistance of titanium, contribute to the outstanding performance of these electrodes. Therefore, TiCu_0.34_ electrodes are promising candidates for long-term health monitoring applications, offering enhanced durability and reliability even in aggressive environments.

## Figures and Tables

**Table 1:** Experimental parameters for plasma treatment used on the activation of the polymeric substrates. (bold is: Polymeric Substrate).

	Atmosphere	Exposure Time (min)	Power (W)	Working Pressure (Pa)
**PU**	Ar	5	50	
**PLA**	Ar + N_2_	15		100
**Cellulose**	O_2_	5		

## Data Availability

The original contributions presented in the study are included in the article, further inquiries can be directed to the corresponding authors.
